# A Vavilovian approach to discovering crop-associated microbes with potential to enhance plant immunity

**DOI:** 10.3389/fpls.2014.00492

**Published:** 2014-09-18

**Authors:** Iago L. Hale, Kirk Broders, Gloria Iriarte

**Affiliations:** ^1^Department of Biological Sciences, University of New HampshireDurham, NH, USA; ^2^Department of Molecular, Cellular, and Biomedical Sciences, University of New HampshireDurham, NH, USA

**Keywords:** metagenomics, domestication, plant breeding, phytobiome, microbiome, inoculants, biocontrol, *in situ* crop wild relatives

## Abstract

Through active associations with a diverse community of largely non-pathogenic microbes, a plant may be thought of as possessing an “extended genotype,” an interactive cross-organismal genome with potential, exploitable implications for plant immunity. The successful enrichment of plant microbiomes with beneficial species has led to numerous commercial applications, and the hunt for new biocontrol organisms continues. Increasingly flexible and affordable sequencing technologies, supported by increasingly comprehensive taxonomic databases, make the characterization of non-model crop-associated microbiomes a widely accessible research method toward this end; and such studies are becoming more frequent. A summary of this emerging literature reveals, however, the need for a more systematic research lens in the face of what is already a metagenomics data deluge. Considering the processes and consequences of crop evolution and domestication, we assert that the judicious integration of *in situ* crop wild relatives into phytobiome research efforts presents a singularly powerful tool for separating signal from noise, thereby facilitating a more efficient means of identifying candidate plant-associated microbes with the potential for enhancing the immunity and fitness of crop species.

## Introduction

The two basic crop improvement strategies available to agricultural scientists are traditionally understood to be: (1) assembling/developing plant genetic diversity and selecting for superior plant genotypes; and (2) modifying growing conditions, primarily via cultural practices, to optimize the desired performance of those superior genotypes. While there is a wide array of cultural practices with potential to significantly influence disease development (e.g., crop rotation, irrigation, pesticide application, etc.), genetic strategies of disease resistance have typically had a much narrower entry point, namely exploitable variation within the host genome itself. The history of plant breeding, therefore, has been punctuated by advances in methods for increasing selectable host genetic diversity, e.g., mutation breeding, embryo rescue to facilitate wide crosses, grafting, the re-creation of ancient polyploids (synthetics) to recover genetic diversity from crop wild ancestors, and transgenic methods to tap useful genetic diversity even beyond the plant kingdom, to name a few. This historic focus on host genome manipulation is understandable given the fact that, to a first approximation, a plant behaves as a single, bounded, coherent organism. However, insights from the maturing field of microbiome ecology indicate that what we perceive to be single organisms, whether animals (Turnbaugh et al., 2007) or plants (Bulgarelli et al., 2013), are in fact complex interacting communities of organisms, with a collective genome size at least an order of magnitude larger than the host genome itself (Turner et al., 2013a). Thus, in addition to modifying the host genome and the growing environment, modifying the plant-associated microbiome, or phytobiome, represents an intriguing complementary strategy for crop improvement.

**Figure 1:**
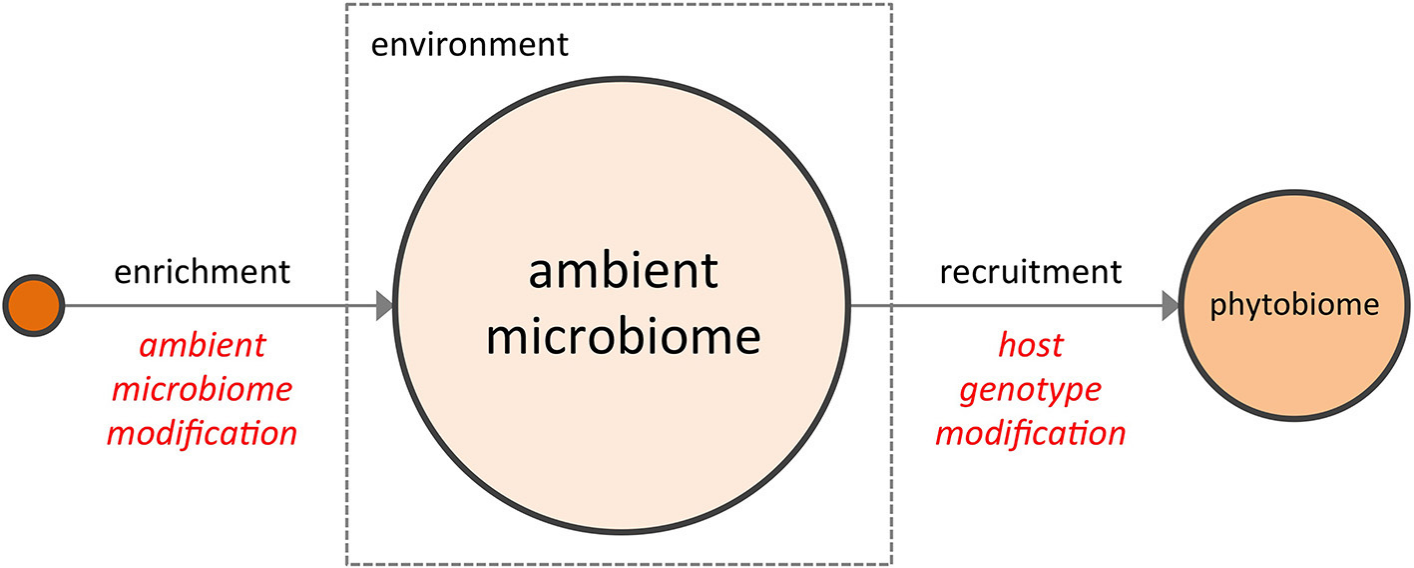
**The two complementary strategies for enhancing a crop phytobiome are direct modification of the ambient microbiome (e.g., the BSM, via inoculants or soil transfers) and the development of host genotypes better able to recruit a superior microbial assembly from the ambient microbiome**.

The notion that the phytobiome can somehow be manipulated to improve crop performance is not new. From biodynamic preparations (Carpenter-Boggs et al., 2000; Zaller and Köpke, 2004) to bulk soil transfers (Weller et al., 2002) to the more conventional and now widespread use of commercial inoculants comprised of plant growth promoting rhizobacteria or pathogen-suppressing microbes, some value has long been recognized in supplementing a plant's ambient microbiome, e.g., the bulk soil microbiome (BSM), with favorable microorganisms to enhance plant performance. More recently, as research has focused on the interaction of the rhizosphere (Costa et al., 2006; Chaparro et al., 2013) with the BSM, interest has grown in the development of disease suppressive soils (Penton et al., 2014), a topic reviewed extensively in the recent literature (Singh et al., 2004; Raaijmakers et al., 2009; Bakker et al., 2012; Berendsen et al., 2012). In its variety of forms, direct enrichment of the ambient microbiome to shift its composition represents one possible means of modifying the crop phytobiome (Figure 1).

In addition to the reservoir of available microbial diversity present within the ambient microbiome, the selectivity of the host plant must also be taken into account. To date, phytobiome research has focused largely on the bacterial communities of the rhizosphere (Marschner et al., 2004; Costa et al., 2006; Paterson et al., 2007; Berg and Smalla, 2009), with relatively less but growing emphasis on phyllosphere and plant-associated fungal communities. While the rhizosphere comprises but a subset of the microbes within the phytobiome, recent metagenomics studies which examined the root endophyte microbiome, the rhizosphere microbiome, and the BSM (Bulgarelli et al., 2012; Lundberg et al., 2012) provide three important and potentially general insights into the phytobiome: (1) significant variation was found among the BSM compositions of geographically distinct soils (i.e., environmental conditions matter); (2) many microbes abundant in the BSM were not found within the rhizosphere or the root endophytic communities (i.e., host plants are selective); and (3) some compositional features of the rhizosphere and root endophytic communities varied, in a repeatable manner, according to plant genotype (i.e., host genotype matters). In other words, the emerging literature on phytobiomes indicates that plants actively influence the assembly of their associated phytobiomes through a non-random, genotype-dependent recruitment of members from the highly-variable ambient microbiome (Aira et al., 2010; Bouffaud et al., 2012; Peiffer et al., 2013), a conclusion supported by phyllosphere studies as well (Knief et al., 2010; Redford et al., 2010). Consequently, a second potential means of modifying the crop phytobiome may lie in selecting host genotypes better able to recruit a superior community of microorganisms from the ambient microbiome (i.e., to assemble a superior “extended genotype”) (Figure 1).

In this perspective, we argue that the practical advancement of either of these potential strategies, ambient microbiome enrichment or host genotype modification, could benefit from the adoption of a research framework which takes into account the processes and consequences of crop evolution and domestication. In particular, the theory of the centers of origin of cultivated plants, first posited by the seminal Russian plant scientist Vavilov (1926), provides a compelling organizing principle in the search for plant-associated microbes with potential to enhance crop fitness and immunity.

## Prospecting for fitness-enhancing crop-associated microbes

Historically, applied research into the microbe-mediated control of plant pathogens relied upon generic culturing methodologies to detect fungi and bacteria with the capacity to exclude or limit a target pathogen via parasitism, competition, and/or antibiosis. Some microorganisms discovered in this way and subsequently ushered through the product development and commercialization pipeline, most notably several species of the fungi *Trichoderma* and *Gliocladium* as well as bacterial species of *Pseudomonas* and *Bacillus*, have proven useful in a wide range of agricultural and horticultural crops (see McSpadden-Gardener and Fravel, 2002; Cawoy et al., 2011; Woo et al., 2014). Recent metagenomic studies of the rhizosphere (Peiffer et al., 2013; Turner et al., 2013b; Donn et al., 2014) and phyllosphere (Lopez-Velasco et al., 2013; Ottesen et al., 2013; Perazzolli et al., 2014) indicate that there is a large, untapped diversity of potentially beneficial microbes existing naturally on and within plants. In terms of prospecting for novel fitness-enhancing microbes, one criticism of culture-based screens is that they exclude the possibility of discovering biocontrol agents not amenable to generic culturing methods. Further, since complex communities of microbes likely work in concert, rather than singly, to buffer both biotic and abiotic stresses (Whipps, 2001; Barea et al., 2005; Hunter et al., 2014), diversity studies based upon culture-independent methods emerged as the new standard entry point for researching the phytobiome.

The evolution of such diversity studies has been swift over the past decade, as the research community transitioned to culture-independent methods such as denaturing gradient gel electrophoresis (DGGE), terminal restriction fragment length polymorphism (t-RFLP), and Sanger sequencing of 16S rRNA fragments. This was followed by the first wave of metagenomic studies using next-generation sequencing (NGS) technologies, primarily 454 pyrosequencing for both bacterial and fungal communities. This approach has now largely been supplanted by a rapidly developing suite of increasingly robust and affordable NGS technologies. The data generated with such technologies is flooding in, as massively parallel sequencing efforts have been undertaken to document the plant-associated microbial communities for a number of agriculturally important plant species.

While metagenomics has revolutionized our conception of the immense taxonomic dimensionality of the phytobiome, it is worth noting that the focus of the crop-associated metagenomic research reported over the past five years (Supplementary Table 1) has primarily been limited to the documentation of microbial diversity (genus-level and higher) associated with the different tissues of a narrow set of plant genotypes, often just one, grown in contrasting locations/treatments. Many of these studies were limited in their ability to detect rare individuals or discriminate among closely-related, but potentially functionally-distinct, species due to shortcomings in the sequencing technologies themselves, including low sequencing depth per unit cost and reliance upon 16S rRNA fragments for taxonomic classification. Significantly, both of these limitations are being overcome by ever-improving sequencing platforms that now provide affordable whole genome metagenomic sequencing. Through longer reads, diminishing costs, and enhanced multiplexing, such platforms enable a substantial increase in both experimental scope and taxonomic resolution.

## Mining the phytobiome—experimental design considerations

The range of potential testable hypotheses motivated by the patterns observed within plant-associated metagenomic data is immense, even leading to favorable comparisons with Darwin's *Voyage of the Beagle* (Gilbert et al., 2011a); but to embark on this journey requires a transition from relatively high-level taxonomic cataloging of phytobiomes to using metagenomics in the service of hypothesis-driven research. Now that highly multiplexed sequencing has become affordable, even for small research programs, we are entering an era in which agricultural researchers are justified in contemplating the use of metagenomics within the context of application-oriented experimental designs.

In terms of general considerations for metagenomic studies, previous reviews have stressed the importance of both biological and technical replication to allow for robust statistical analysis; and standards for metadata collection will certainly improve comparisons among datasets (Field et al., 2008). Broad, shallow, replicated sequencing across large numbers of samples, followed by targeted deep sequencing and single-cell genomics enable investigators to define and refine hypotheses, detect samples most likely to provide insight, and then identify metabolic potential within single genomes up to the community as a whole (Knight et al., 2012). Such general strategies will be of particular importance for agricultural and plant scientists as they begin to investigate the effects of crop genotype, crop phenostage, cultural practices, soil type, temperature, and a host of other potentially important environmental variables on the phytobiome.

In terms of more specific considerations, a research program committed to developing practical improvements in plant fitness via phytobiome modification confronts three fundamental issues:

1. *Breeders successfully select for traits with unknown underlying mechanisms*. The power of phenotypic selection lies in the fact that one can develop superior crop genotypes without having any understanding of the genetic mechanisms underlying their superiority. For example, to identify and select a plant exhibiting immunity under field conditions is relatively straightforward and requires no knowledge of the basis of that immunity, be it a novel resistance gene, a pyramid of genes of minor effect, an expression polymorphism, a more robust phytobiome (extended genotype), etc. Thus, it is likely that humans already have been selecting, however inadvertently, for those regions of the host genome which improve phytobiome recruitment, to the extent that such recruitment is both heritable and enhances fitness. Is it therefore possible for a research program to find novel, favorable (i.e., fitness-enhancing) microbial associations missed by human selection?
2. *It is hard to beat natural selection*. To the extent that the plant characteristics we are interested in improving are related to individual fitness, which is certainly the case with immunity to disease, such traits have likely been subjected to natural selection over evolutionary timescales. Similarly, to the extent that plants actively forge associations with beneficial microbes, such associations may themselves be the results of long co-evolutionary histories (Martínez-Romero, 2009). Applying Denison et al.'s (2003) insightful “Darwinian agriculture” concept to this question of phytobiome modification then begs the question: What opportunities exist for tradeoff-free, phytobiome-mediated crop improvement that were missed by natural selection?
3. *Crop plants cannot associate with absent microbes*. Over the past 10 years, great strides have been made in determining global patterns of microbial diversity in a number of terrestrial (Fierer and Jackson, 2006; Lauber et al., 2009) and aquatic (Newton et al., 2007; Gilbert et al., 2011b) environments. While soil bacterial biomes of different environmental and edaphic provenances may share patterns of compositional diversity in terms of their dominant phyla (Fierer et al., 2009), it is clear at the level of operational taxonomic units that significant BSM diversity can exist, even among related sites (Lundberg et al., 2012). And given that related members within a single bacterial or fungal genus can vary widely in terms of the nature of their interactions with a host plant genotype, ranging from endophytic to pathogenic (Slippers and Wingfield, 2007; Junker et al., 2012), it is evident that a robust research strategy requires a non-arbitrary means of dealing with the pervasive issue of BSM (i.e., ambient microbial) variability.

## Domestication, migration, and the promise of *in situ* crop wild relatives

As one means of addressing the three fundamental issues outlined above, it is instructive to consider that all of our important crop species today were domesticated from wild ancestors, sometimes over periods of thousands of years (Gepts, 2004), in specific geographic regions first hypothesized by Vavilov (1926) and variously referred to as “centers of diversity” or “centers of domestication.” While the exact number and extent of these centers continue to be debated (Smith, 2006), Vavilov's essential premise endures. Following domestication, landraces were often grown in these same centers of domestication alongside, and likely intermating with (Ellstrand et al., 1999), wild populations as well as transported to new regions by early agriculturalists (Denham, 2013) or non-human vectors (Erickson et al., 2005). In the era of modern breeding, such landraces frequently served as the base germplasm for further crop improvement (Harlan, 1975), resulting in cultivated varieties that are now grown around the globe (i.e., far from their centers of origin).

One well-documented consequence of this history of crop improvement is the so-called founder effect (Simmonds, 1976; Ladizinsky, 1985), in which the genetic diversity present within modern, adapted crop germplasm is substantially lower than in its wild relatives due to the genetic bottlenecks imposed by the processes of domestication and breeding. For this reason, plant breeders have long viewed crop wild relatives as an invaluable source of potentially useful “lost” genetic diversity; indeed, there are numerous examples of adapted cultivars benefiting from the strategic introgression of wild genes, particularly in cases of disease resistance (Hajjar and Hodgkin, 2007).

Another potential consequence of this history of crop improvement, and one that may have particular relevance to the question of exploitable microbial associations, is the fact that modern crop varieties, grown far from their centers of origin under agricultural conditions, necessarily assemble their associated phytobiomes from *ex situ* ambient microbiomes that likely differ from the *in situ* ambient microbiomes with which their wild relatives co-evolved. Just as the processes of domestication and breeding resulted in lost host diversity due to genetic bottlenecks, the process of migration may have resulted in lost associated microbial diversity due to a physical dislocation of host plant genotypes from their co-evolved microorganisms.

## Common gardens, uncommon harvests

In light of the arguments outlined above, *in situ* crop wild relatives (*is*CWR's) may be the best available bio-assays for lost, co-evolved microbial diversity with the potential to confer enhanced fitness on our crop species. And importantly, a research program focused on characterizing *is*CWR phytobiomes successfully addresses the three fundamental issues intrinsic to applied phytobiome research, because:

1. *Modern breeding could have missed it*. Since modern, adapted varieties were selected in target agricultural environments usually far from their centers of diversity, a focus on the phytobiomes of wild germplasm in non-agricultural *in situ* soils makes it possible to discover beneficial microbial associations missed by plant breeders.
2. *It passes the “Darwinian agriculture” challenge*. Since migration physically disrupts the associations between host plants and their co-evolved cohorts of environment-specific ambient microorganisms, probing *is*CWR's for lost beneficial microbial associations is, in essence, an attempt to re-capture and leverage, rather than outdo, naturally-selected and vetted associations.
3. *Ambient in situ microbial communities are co-evolutionarily meaningful*. While the ambient microbiomes alive today in undisturbed sites within crop centers of diversity are not equivalent to those present during the evolution of our crop wild ancestors, they may be the best proxy of the latter available to us. Therefore, in the search for untapped microbial associations able to confer tradeoff-free increases in crop fitness, *in situ* ambient communities of microorganisms provide a reasonable, and non-arbitrary, focus.

**Figure 2:**
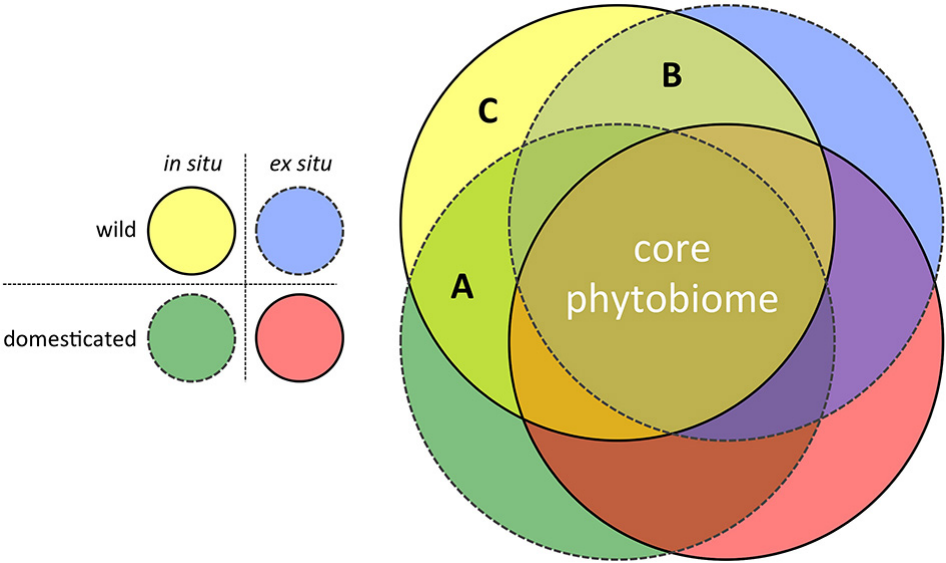
**Characterizing the phytobiome composition of replicated common gardens, consisting of wild and domesticated/improved genotypes, grown in both the center of diversity (*in situ*) and under agricultural conditions outside the center of diversity (*ex situ* target environments), has the potential to identify the effects of both domestication and migration on the disruption of beneficial co-evolved crop-microbe associations**. **(A)** Lost microbial associations due to migration of the host from the center of diversity (i.e., microbial associations still forged by modern crop genotypes, if the respective microbes are present = an inoculation opportunity); **(B)** Lost microbial associations due to host domestication (i.e., modern crop genotypes have lost the ability to forge these associations, even when the respective microbes are present = a breeding opportunity); and **(C)** Lost microbial associations due to both migration and domestication (i.e., both breeding and ambient microbiome modification would be required to restore these associations).

In terms of experimental design, the move toward such hypothesis-driven metagenomics research for applied crop phytobiome improvement can assume a simple form: Replicated common gardens, containing a random selection of wild and adapted (modern) genotypes, grown in multiple locations both *in situ* (i.e., non-agricultural sites within centers of diversity) and *ex situ* (i.e., target agricultural environments). The power of this approach to efficiently identify those potentially rare microorganisms lost to our modern agricultural systems due to the effects of both domestication and migration is illustrated in Figure 2. In the search for novel microorganisms with the potential to enrich the crop phytobiomes, three regions in the figure are of particular interest:

- Microbes detected in the phytobiomes of plants grown *in situ* but not *ex situ*. This region of the diagram contains those microbial species that are candidates for inoculation (i.e., *ex situ* ambient microbiome enrichment) because modern crop genotypes retain the ability to associate with them, if they are present.
- Microbes detected in crop wild relatives but not improved genotypes. This region of the diagram contains those microbial associations that are candidates for breeding. Perhaps due to drift or tight linkage to genes related to the domestication syndrome, modern crop genotypes have lost the ability to associate with these microbial species, despite their presence in the ambient microbiome.
- Microbes only detected in association with *is*CWR's. This region of the diagram contains those microbial species lost to current agricultural systems due to the combined effects of domestication (breeding) and migration. Restoring associations with such microbes would likely be more difficult, requiring both host genotype modification and ambient microbiome enrichment.

Of the three regions in the figure, region A would be of most interest in the context of identifying a subset of candidate microbes within the highly diverse phytobiome with potential application as novel biocontrol organisms and fitness-enhancing inoculants.

## What would vavilov sequence?

In the face of the emerging crop metagenomics data deluge, a conceptual framework is needed to parse signal from noise in the hunt for promising directions in the field of applied crop phytobiome research. As argued in this perspective, the strategic use of *is*CWR's offers one guide through the complexity, facilitating the efficient identification of candidate plant-associated microbes with reasonable potential to enhance the fitness of crop species. In other words, though it is perhaps counter-intuitive, one strategy for tackling the growing abundance of largely descriptive crop metagenomics data is to sequence *more* phytobiomes, specifically those of *is*CWR's, since such datasets provide lenses through which the full dimensionality of the phytobiome may be dramatically reduced, as illustrated in Figure 2.

For those crops whose centers of domestication are relatively well-characterized and for which extensively characterized germplasm collections exist (e.g., common bean, wheat, maize, potato, coffee, etc.), the research direction proposed here is relatively straightforward. For those with a history of relatively less investment in domestication research and diversity collection/assessment, the potential of this kind of investigation to improve agricultural productivity underscores the practical importance of basic crop domestication research. Beyond this, it highlights a categorical difference between *ex situ* germplasm conservation (e.g., seed banks, repositories, etc.) and *in situ* germplasm conservation (e.g., conservation areas in centers of diversity, landrace maintenance in centers of domestication, etc.). While the former may succeed in preserving host genetic diversity for use by plant breeders, only the latter enables exploration of the approximate microbial co-evolutionary context of our crop wild ancestors. Thus, to the extent that ambient microbiome enrichment has any potential to boost crop plant immunity under field conditions, the value of *in situ* germplasm conservation should not be underestimated.

As potentially useful as *is*CWR's may prove to be as bio-assays for microbial associations of co-evolutionary significance, it is worth noting that any candidate microbes, individuals, or groups identified via metagenomics, even in this directed manner, are little more than that: candidates. Each NGS sequencing platform, though differing in upstream preparations and downstream analyses, has the potential to detect and differentiate rare microbes in the phytobiome; but none can differentiate live from dead cells, nor meaningful associations from coincidental ones. Thus, misinterpretations of the data, including diversity overestimations and pattern significances, are likely. As with all metagenomics studies, it will therefore be key to follow up initial sequence-based phytobiome parsing with concerted efforts to determine which species are present and to develop improved methods for culturing a greater number of these organisms from the host tissue as we endeavor to move beyond measures of diversity to an understanding of exploitable functions of the bacteria and fungi inhabiting our crop plants.

### Conflict of interest statement

The authors declare that the research was conducted in the absence of any commercial or financial relationships that could be construed as a potential conflict of interest.
